# Transcriptional patterns of brain structural abnormalities in CSVD-related cognitive impairment

**DOI:** 10.3389/fnagi.2024.1503806

**Published:** 2024-11-29

**Authors:** Haixia Mao, Min Xu, Hui Wang, Yuankun Liu, Feng Wang, Qianqian Gao, Songyun Zhao, Lin Ma, Xiaoyun Hu, Xiaoxuan Zhang, Guangjun Xi, Xiangming Fang, Yachen Shi

**Affiliations:** ^1^Department of Radiology, The Affiliated Wuxi People’s Hospital of Nanjing Medical University, Wuxi People’s Hospital, Wuxi Medical Center, Nanjing Medical University, Wuxi, China; ^2^Department of Neurology, The Affiliated Wuxi People’s Hospital of Nanjing Medical University, Wuxi People’s Hospital, Wuxi Medical Center, Nanjing Medical University, Wuxi, China; ^3^Department of Interventional Neurology, The Affiliated Wuxi People’s Hospital of Nanjing Medical University, Wuxi People’s Hospital, Wuxi Medical Center, Nanjing Medical University, Wuxi, China; ^4^Department of Neurosurgery, The Affiliated Wuxi People’s Hospital of Nanjing Medical University, Wuxi People’s Hospital, Wuxi Medical Center, Nanjing Medical University, Wuxi, China

**Keywords:** small cerebral vascular disease, cognitive impairment, subcortical vascular cognitive impairment, gray matter volume, transcription, Allen Human Brain Atla

## Abstract

**Background:**

Brain structural abnormalities have been associated with cognitive impairment in individuals with small cerebral vascular disease (CSVD). However, the molecular and cellular factors making the different brain structural regions more vulnerable to CSVD-related cognitive impairment remain largely unknown.

**Materials and methods:**

Voxel-based morphology (VBM) was performed on the structural magnetic resonance imaging data of 46 CSVD-related cognitive impairment and 73 healthy controls to analyze and compare the gray matter volume (GMV) between the 2 groups. Transcriptome-neuroimaging spatial correlation analysis was carried out in combination with the Allen Human Brain Atlas to explore gene expression profiles associated with changes in cortical morphology in CSVD-related cognitive impairment.

**Results:**

VBM analysis demonstrated extensive decreased GMV in CSVD-related cognitive impairment in the bilateral temporal lobe and thalamus, especially the hippocampus, thalamus, parahippocampus, and fusiform, and the left temporal lobe showed a more severe atrophy than the right temporal lobe. These brain structural alterations were closely related to memory and executive function deficits in CSVD-related cognitive impairment. Furthermore, a total of 1,580 genes were revealed to be significantly associated with regional change in GMV. The negatively and positively GMV-linked gene expression profiles were mainly enriched in RNA polymerase II, catalytic activity acting on a nucleic acid, aminoacyltransferase activity, axonogenesis, Golgi membrane, and cell junction organization.

**Conclusion:**

Our findings suggest that brain morphological abnormalities in CSVD-related cognitive impairment are linked to molecular changes involving complex polygenic mechanisms, highlighting the interplay between genetic influences and structural alterations relevant to CSVD-related cognitive impairment.

## Introduction

CSVD is a progressive disease and is associated with a decline in a wide range of cognitive abilities (Tap et al., 2023), such as memory disorders, executive function impairment, and attention disturbances (Bhat et al., 2022; Hotz et al., 2023) which may continue to worsen as the CSVD progresses. CSVD-related cognitive impairment accounting for > 50% of vascular dementia and associated with a higher dementia risk (Kalaria and Erkinjuntti, 2006). However, the underlying neuropathological mechanism of cognitive impairments in CSVD patients is unclear. The development of CSVD is influenced by a complex mix of genetic and environmental risk factors, and the current primary approach to cure this disease is to control vascular risk factors such as hypertension, dyslipidemia, diabetes, and smoking (Inoue et al., 2023). However, the cascade of events and molecular pathways that lead to the alterations in the structure and function of CSVD are largely unknown. Molecular biomarkers for clinical use have not been identified and, besides risk factor management (for example blood pressure), no mechanism-based treatments are available to prevent CSVD progression and complications. Therefore, the exploration of neuropathological mechanisms regulating the cognitive impairment caused by CSVD is particularly important for early prevention and treatment of CSVD-related cognitive impairment.

CSVD causes widespread brain gray matter (GM) damage, mostly becoming atrophic (Zhu et al., 2021; da Silva et al., 2023; Li et al., 2023). Studies have recently focused on the interplay among CSVD, medial temporal lobe degeneration, and cognitive decline (Perosa et al., 2020; Mennen et al., 2024). The cognitive profile of the CSVD cohort is, moreover, consistent with a concomitant deleterious effect on the medial temporal lobe, which includes deficits in episodic memory, a cognitive domain strongly related to hippocampal function (Liu et al., 2020). The current consensus is that CSVD patients are characterized by an extensive degeneration of GM structure, especially in the bilateral temporal lobe and hippocampus, which is closely associated with cognitive impairment (Banker et al., 2021). However, the human cerebral cortex possesses a tremendous cellular diversity (Fang et al., 2022); thus, despite growing evidence linking brain morphology with cognitive deficits in CSVD-related cognitive impairment, the underlying molecular and cellular mechanisms that predispose specific brain regions to structural alterations remain inadequately understood.

Epigenetic mechanisms are involved in primate brain development and aging (Ning et al., 2024), which cause the change in brain connectomes, which in turn is influenced by genetic factors, as confirmed by many studies (Zhong et al., 2021; Moreau et al., 2023; Dong et al., 2024); brain-wide gene expression atlases bridge the gap between connectomes and transcriptomes (Arnatkeviciute et al., 2023; Estevez-Fraga et al., 2023). The Allen Human Brain Atlas (AHBA) microarray dataset has been used to identify transcriptomes associated with human neuroimaging (Zhu et al., 2024) with multimodal evidence suggesting a link between conserved gene expression and functionally relevant circuitry (Li et al., 2024). The combination of neuroimaging with gene transcripts provides insight into the ability of disease-related alterations at the microscale architecture to drive macroscale brain abnormalities in various diseases (Luo et al., 2023).

Our hypothesis is that CSVD-related cognitive impairment, caused by GMV alterations, is genetically related at a molecular level. Therefore, this study explores the link between CSVD-related cognitive impairment, GM volume abnormalities, and transcriptional data to improve understanding of how molecular and cellular mechanisms relate to structural changes in this condition. Firstly, the VBM method was used to detect the alteration of GM structure. Subsequently, spearman correlation and partial least squares regression analysis based on the brain-wide gene expression of AHBA microarray dataset were performed to assess the relationship between identified GM degeneration pattern and neurotransmitter/transcriptome. Finally, a functional enrichment analysis was conducted to understand the ontological pathways of genes associated with CSVD-related cognitive impairment. Our findings may help improve understanding of how gene expression, cell types, and molecular functions throughout the brain influence the anatomical changes observed in CSVD-related cognitive impairment.

## Materials and methods

### Participants

Patients in the CSVD-related cognitive impairment group were recruited from the Department of Neurology of the Affiliated Wuxi People’s Hospital of Nanjing Medical University between December 2022 and September 2023. Healthy controls (HCs) matching gender, age, and education level were recruited at the same time. All subjects were subjected to MRI scans and a comprehensive neuropsychological evaluation.

The patients with CSVD-related cognitive impairment met the following criteria: age 50–80 years; education level of more than 6 years; no history of malignant tumor, craniocerebral operation, massive cerebral infarction (greater than 15 mm in size), cerebral hemorrhage, other serious systemic disorders, or mental illness; no MRI contraindications; MRI findings consistent with neuroimaging standards for research into small vessel disease include recent small subcortical infarct (infarction in the territory of one perforating arteriole), lacune (presumed vascular origin, cavity up to 15 mm in diameter), white matter hyperintensity (presumed vascular origin; subcortical gray matter or brainstem not included), perivascular space (fluid-filled space, diameter commonly not exceeding 2 mm when imaged perpendicular to the vessel), cerebral microbleed (usually 2–5 mm, sometimes up to 10 mm), cortical superficial siderosis, brain atrophy, and cortical cerebral microinfarct (small lesions, strictly cortical with an upper size limit of 4 mm) (Duering et al., 2023); complaint of cognitive impairment from the patients or caregivers persisting at least 3 months and neuropsychological assessment results meeting the VICCCS-2 protocol for VCI diagnosis (Skrobot et al., 2018). The exclusion criteria were the following: clinical large intracranial vascular disorders, other causes of cognitive impairment (e.g., Alzheimer’s disease), and MRI images with artifacts affecting the image quality.

All HCs had normal results in neurological examinations, including neuropsychological assessments and MRI (please see Supplementary Figure 1), with no history of mental disorders or major systemic diseases affecting the heart, lungs, liver, or kidneys.

### Neuropsychological assessment

Each patient was subjected to a global cognition and four cognitive domain tests. The four cognitive domain tests contained executive functions, information processing speed, as well as memory and visuospatial functions. The detailed rating scale is shown in the Supplementary material. Participants with Montreal Cognitive Assessment below 25 were classified as having a cognitive impairment (Gil-Berrozpe et al., 2020). All raw data were transformed into standard scores (z-scores), which were averaged to assess the general cognitive function and other cognitive domains (Shi et al., 2023; Shi et al., 2024). The executive function scores were the average of TMT-B, Stroop-C, and DST-backward scale (Z scores) scores, the information processing speed scores were the average of TMT-A, Stroop-A, and Stroop-B scales (Z scores) scores, and the memory function total scores were the average of the AVLT-IR and AVLT-20 min DR (Z scores) scores.

### MRI data acquisition

MRI data from all subjects were obtained from a 3.0-Tesla MR system (Prisma, Siemens Medical Solutions, Inc., Germany). Head motion was minimized using foam padding. Participants were instructed to stay still, close their eyes, and stay awake during the scan. Three-dimensional T1-weighted images (3D_T1WI) followed by the acquisition of T2-weighted imaging (T2WI), T2WI fluid-attenuated inversion recovery (T2WI-FLAIR), susceptibility-weighted imaging (SWI), three-dimensional time-of-flight (3D_TOF) and diffusion weighted imaging (DWI) were obtained to identify and remove any subtle brain lesions that were not clinically evident. 3D-T1WI scans were acquired with the following parameters: repetition time (TR) = 2,000 ms, echo time (TE) = 2.49 ms, flip angle (FA) = 9°, field of view (FOV) = 256 mm × 256 mm, matrix size = 256 × 256, slice thickness = 1 mm with no gap, and 192 sagittal slices.

The disease was diagnosed, and other brain lesions were excluded, by three senior radiologists with over five years of experience in radiodiagnosis. Additionally, each radiologist independently assessed the summary CSVD score (Duering et al., 2023) for each patient. In cases of inconsistency, a consensus was reached through discussion and mutual agreement.

### VBM analysis

The spm12 software operating in the MATLAB R2019b environment, was used for the preprocessing and segmentation of 3D_T1WI data. First, the structural images were segmented into gray matter (GM), white matter, and cerebrospinal fluid, and then further normalized to the Montreal Neurological Institute (MNI) standard space. Second, the GM normalized images were modulated to correct for volume changes during the normalization process. Third, spatial smoothing was performed using a Gaussian kernel with a full width at half maximum (FWHM) of 6 mm, followed by statistical analysis after checking image quality. Finally, an independent 2-sample *t*-test was conducted to identify brain regions with significant case-control differences in GMV. The significance level was determined using family-wise error (FWE) correction, with a voxel-wise *p* < 0.05.

### Correlation analysis

Correlation analysis was carried out between GMV of regions with statistical differences and neuropsychological assessment to explore whether neuroimaging indices were related to the cognitive function of CSVD-related cognitive impairment. The significance level was set at *p* < 0.05.

### Brain gene expression data processing

The AHBA^1^ provides normalized microarray expression data from six donated human brains, including more than 20,000 genes across 3,702 samples of brain tissue (Li et al., 2021; Qin et al., 2024). The gene expression data were preprocessed using the abagen toolbox.^2^ The detailed preprocessing steps are listed in the Supplementary material. The Automated Anatomical Labeling (AAL) brain atlas was used to parcellate the brain to acquire the gene expression matrix. AAL divided the brain into 116 brain nodes, including 45 in the left hemisphere, 45 in the right hemisphere, and 26 in the cerebellum, and was spatially matched with 15,633 gene expression profiles to obtain a 116 × 15,633 matrix. Only the left hemisphere was considered in our analysis since the AHBA dataset includes two right hemisphere data points alone (Li et al., 2021). Thus, a mean of all samples in a region was calculated to obtain the matrix (45 regions × expression of 15,663 genes) of transcriptional level values.

### Spatial correlation analysis of gene expression neuroimaging

The genes mostly correlated with GMV changes in CSVD-related cognitive impairment were obtained by spatially matching the statistical maps of GMV with the 45 left hemisphere nodes of the AAL brain atlas, and their average weights were further calculated. Partial least squares (PLS) regression (Colombani et al., 2012) was used to determine the relationship between regional changes in GMV (*t*-values from 45 cortical regions in the left hemisphere) and the transcriptional activity of all the 15,633 genes. Gene expression data were used as predictor variables of regional changes in GMV in the PLS regression. The first component (PLS1) was defined as the spatial map that captured the greatest fraction of total gene expression variance across cortical areas (Li et al., 2021) and represented the linear combination of gene expression values that showed the strongest correlation with regional changes in GMV. The permutation testing (5,000 times) based on spherical rotations of the GMV map to account for spatial autocorrelation was used to test the null hypothesis that PLS1 explained no more covariance between the GMV map and whole-genome expression than would be expected by chance (Romero-Garcia et al., 2020). Bootstrap resampling was used to assess the variability of PLS1 for each gene, and the ratio between the weight of each gene and its bootstrap standard error was used to determine Z scores. Subsequently, normalized weight genes were ranked based on Z scores, which represented the gene respective contribution to PLS1 (Morgan et al., 2019). The gene sequences of the top 5% and the bottom 5% Z scores (normalized PLS1 weights) were extracted as positive (PLS1+, Z score range from 1.85 to 5.63) or negative (PLS1−, Z score range from −4.65 to −1.50) gene sets associated with regional changes in GMV.

### Enrichment analysis

Metascape analysis^3^ offers automated meta-analysis tools to explore common or unique pathways across 40 independent knowledge bases (Zhou et al., 2019). The PLS1+ and PLS1− genes were submitted into the Metascape website, and the significance of the resulting enrichment pathways was assessed at a threshold of 5%, adjusted for false discovery rate (FDR). Four terms, such as gene ontology (GO) biological processes, GO Cellular Components, GO Molecular Functions, and Kyoto Encyclopedia of Genes and Genomes (KEGG) pathway, were considered to obtain the biological explanation for CSVD-related cognitive impairment. All genes in the genome were as the enrichment background. Terms with a value of *p* < 0.05, a minimum count of 3, and an enrichment factor > 1.5 (the enrichment factor is the ratio between the observed counts and the counts expected by chance) were collected and grouped into clusters based on their membership similarities.

### Statistical analysis

Statistical analysis was performed using SPSS 26.0. Continuous variables with a normal distribution were expressed as mean ± standard deviation (SD), while variables with a non-normal distribution were expressed as median with interquartile range. The Chi-squared (χ^2^) test was used for categorical data, two-sample *t*-test was used for the comparison between two groups with normally distributed data, and the Mann–Whitney U test was used for the comparison between non-normally distributed data. A value of *p* < 0.05 was considered statistically significant.

## Results

### Demographic and clinical characteristics

The CSVD-related cognitive impairment group included 46 patients (17 males, 29 females; mean age: 70.26 ± 4.11 years). Seventy-three HCs matching for sex, age, and educational level, were recruited (26 males, 47 females; average age: 68.81 ± 3.80 years). No statistically significant difference was found in age (*p* = 0.051), sex (*p* = 0.882), or education level (*p* = 0.106) between CSVD-related cognitive impairment and HCs, while a statistically significant difference was found in cognitive function (Table 1).

**TABLE 1:** Demographic and clinical characteristics.

Group	CSVD-related cognitive impairment	HC	*p*-value
Sex			0.882*
Male	17	26	
Female	29	47	
Education (years)	9.28 ± 2.46	10.03 ± 2.41	0.106^#^
Age (years)	70.26 ± 4.11	68.81 ± 3.80	0.051^#^
Summary CSVD score	1 (1, 3)	0	< 0.001^&^
MMSE	25.00 (24.00, 25.00)	29.00 (28.00, 30.00)	< 0.001^&^
MoCA	23.00 (19.00, 25.00)	28.00 (27.00, 29.00)	< 0.001^&^
TMT-B (raw score)	266.00 (229.75, 395.75)	133.00 (99.00, 161.00)	< 0.001^&^
TMT-B (z-score)	−0.39 (−0.57, 0.24)	0.05 (−0.83, 0.78)	< 0.001^&^
Stroop-C (raw score)	−0.30 (−0.64, 0.16)	77.53 ± 19.25	< 0.001^&^
Stroop-C (z-score)	151.00 (116.00, 197.75)	−0.18 (−0.78, 0.70)	< 0.001^&^
DST-backward (raw score)	3.50 (3.00, 4.00)	5.00 (4.00, 5.00)	< 0.001^&^
DST-backward (z-score)	0.02 (−0.43, 0.47)	0.73 (−0.68, 0.73)	< 0.001^&^
Executive function	0.27 (−0.08, 0.58)	−0.20 ± 0.25	< 0.001^&^
TMT-A (raw score)	100.00 (80.25, 121.25)	54.00 (43.00, 66.00)	< 0.001^&^
TMT-A (z-score)	−0.16 (−0.64, 0.35)	−0.11 (−0.63, 0.47)	< 0.001^&^
Stroop-A (raw score)	36.00 (30.00, 43.00)	25.00 (22.00, 30.00)	< 0.001^&^
Stroop-A (z-score)	−0.21 (−0.42, 0.05)	−0.19 (−0.81, 0.85)	< 0.001^&^
Stroop-B (raw score)	63.50 (55.75, 75.00)	43.36 ± 11.03	< 0.001^&^
Stroop-B (z-score)	−0.22 (−0.42, 0.09)	−0.12 (−0.80, 0.78)	< 0.001^&^
Information processing speed	0.34 (0.20, 0.76)	−0.40 ± 0.29	< 0.001^&^
AVLT-IR (raw score)	4.21 ± 4.33	6.33 (5.67, 8.00)	< 0.001^&^
AVLT-IR (Z score)	0.00 ± 1.00	−0.19 (−0.58, 0.82)	< 0.001^&^
AVLT-20 min DR (raw score)	2.50 (0.00, 4.25)	6.00 (5.00, 8.00)	< 0.001^&^
AVLT-20 min DR (z-score)	−0.09 (−1.11, 0.63)	−0.18 (−0.61, 0.68)	< 0.001^&^
Memory function	−0.76 ± 0.69	0.48 ± 0.77	< 0.001^#^
CDT (raw score)	9.00 (8.00, 9.00)	9.00 (9.00, 10.00)	< 0.001^&^
CDT (z-score)	0.54 (−0.15, 0.54)	−0.33 (−0.33, 0.88)	< 0.001^&^

HC, healthy control; CSVD, cerebral small vessel disease; MMSE, Mini-mental State Examination; MoCA, Montreal Cognitive Assessment; TMT-B, Trail Making Test B; Stroop-C, Stroop color, and Word Test C; DST-backward, Digit Span Test backward; TMT-A, Trail Making Test A; Stroop-A, Stroop color and Word Test A; Stroop-B, Stroop color and Word Test B; AVLT-IR, Auditory Verbal Learning Test-immediate recall; AVLT-20 min DR, Auditory Verbal Learning Test-20-min delayed recall; CDT, Clock Drawing Test.

*Chi-square test;

^#^Two independent samples *t*-test;

^&^Mann–Whitney U test.

### Changed GMV

**FIGURE 1:**
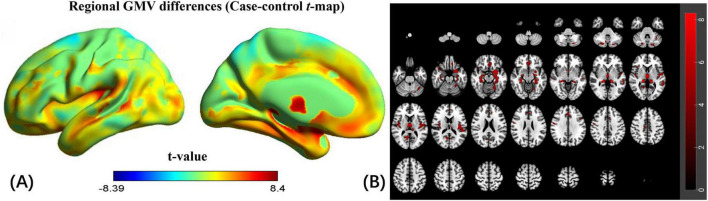
Regional gray matter volume (GMV) changes of CSVD-related cognitive impairment compared to HCs. **(A)** Changes in regional GMV in the left cerebral hemisphere (unthresholded). **(B)** Changes in regional GMV in the whole brain (FEW corrected, *p* < 0.05).

The VBM analysis revealed a significant regional decrease in GMV in the bilateral hippocampus, thalamus, parahippocampus, fusiform, rolandicoper, frontal_med_orb, left amygdala, rectus, temporal_mid, temporal_sup, hesch, and right calcarine and insula in CSVD-related cognitive impairment compared to HCs (Figure 1, family-wise error (FEW) corrected, *p* < 0.05). The detailed information on significantly different regions is listed in the Supplementary Table 1.

### Correlation analysis

**FIGURE 2:**
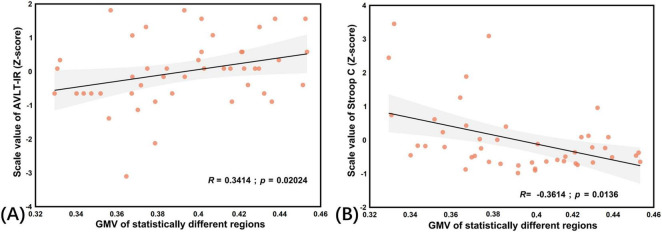
Correlation analysis between GMV of regions with cognitive function. **(A)** Correlation analysis between GMV of regions with statistical differences and scale values of ALVT-IR in CSVD-related cognitive impairment. In CSVD-related cognitive impairment, GMV of regions with statistical differences was positively correlated with memory function. **(B)** Correlation analysis between GMV of regions with statistical differences and scale values of Stroop C in CSVD-related cognitive impairment. In CSVD-related cognitive impairment, The GMV of regions with statistical differences was negative with Stroop C scores (positively correlated with executive function).

The GMV of regions with statistical differences was positively correlated with AVLT-IR scores, while negatively correlated with Stroop-C scores (Figure 2). The GMV of regions with statistical differences was positively correlated with memory function and executive function.

### Transcriptional correlations of changes in GMV related to CSVD-related cognitive impairment

**FIGURE 3:**
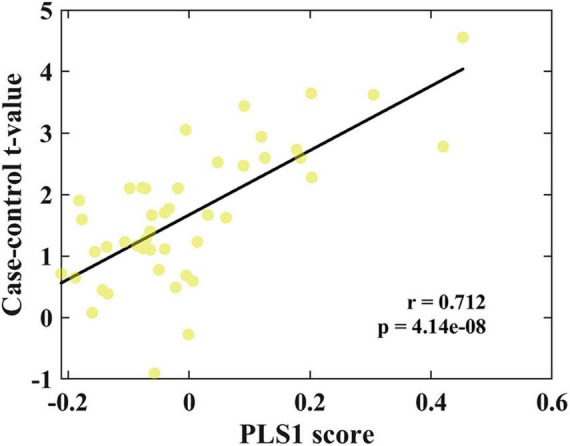
Correlation analysis between PLS1 weighted gene expression map and the case-control *t*-map. Scatterplot of regional PLS1 scores (a weighted sum of 15,633 gene expression scores) and regional changes in GMV (Pearson’s *r*_(150)_ = 0.712, *p*_spin_ < 0.0001).

PLS1 in the discovery cohort explained 39.74% of the variance (Supplementary Figure 2). The distribution of the PLS1 weighted map reflected an anterior-posterior gradient of gene expression (Supplementary Figure 3), which was spatially correlated with the case-control *t*-map (linear regression, *R* = 0.712, *p* < 0.0001; Figure 3). The top 5% and bottom 5% normalized PLS1 weights of the gene sequence were extracted according to the sorted Z-value. A total of 782 positively (PLS1+) correlated genomes and 782 negatively (PLS1−) correlated genomes were found (all *p* < 0.05, FDR corrected). In total, 1,564 genes represented the regional change in GMV gene list in CSVD-related cognitive impairment individuals. The list of genes to be enriched is shown in Supplementary Table 2.

### Pathway and process enrichment analysis

**FIGURE 4:**
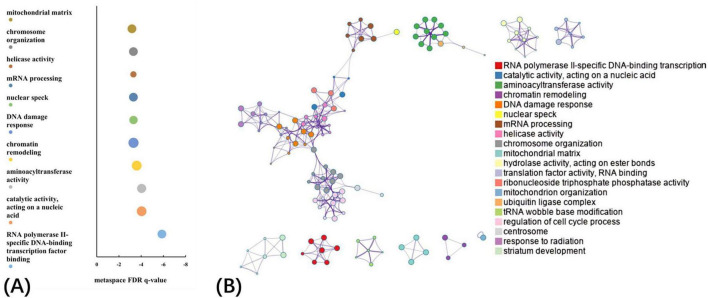
Functional enrichment of PLS1– gene transcripts. **(A)** Top 10 clusters with their representative enriched terms of PLS1– genes. Bubble chart of enriched terms across input gene lists for PLS1– genes, colored by the multi-test adjusted *p*-value in log base 10. The size of the circle represents the number of genes involved in a given term. **(B)** Functional enrichment of gene transcripts of PLS1– genes. Metascape network of enrichment showed cluster similarities of enriched terms. Each term is represented by a circle node, where its size is proportional to the number of input genes included in that term, and its color represents its cluster identity, where nodes that share the same cluster ID are typically close to each other.

**FIGURE 5:**
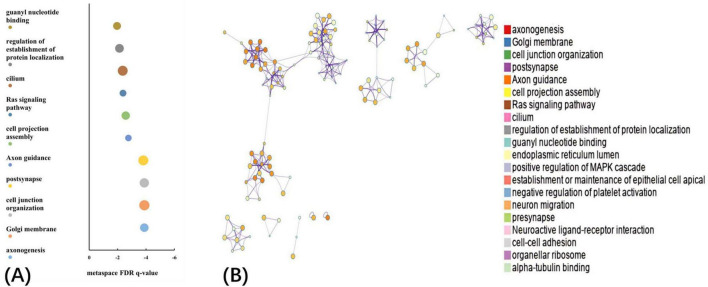
Functional enrichment of PLS1+ gene transcripts. **(A)** Top 10 clusters with their representative enriched terms of PLS1+ genes. Bubble chart of enriched terms across input gene lists for PLS1+ genes, colored by the multi-test adjusted *p*-value in log base 10. The size of the circle represents the number of genes involved in a given term. **(B)** Functional enrichment of gene transcripts of PLS1+ genes. Metascape network of enrichment showed cluster similarities of enriched terms. Each term is represented by a circle node, where its size is proportional to the number of input genes included in that term, and its color represents its cluster identity, where nodes that share the same cluster ID are typically close to each other.

The GO biological processes, GO cellular components, GO molecular functions, and KEGG pathways were aligned with the PLS1− and PLS1+ gene list using Metascape. The results of the PLS1− and PLS1+ gene enrichment analysis were obtained after correcting for enrichment terms (pFDR < 0.05) and discarding discrete enrichment clusters, which are shown in the Supplementary Figures 4, 5, respectively. PLS1− gene enrichment highlighted significantly enriched categories in GO biological processes, GO cellular components, and GO molecular functions. Gene expression profiles were enriched in “RNA polymerase II-specific DNA-binding transcription factor binding,” “catalytic activity, acting on a nucleic acid,” “aminoacyltransferase activity,” “chromatin remodeling,” and “DNA damage response.” No significant enrichment in KEGG pathways was observed. The top ten significant clusters with their representative enriched terms and gene enrichment network visualization of PLS1− genes are shown in Figure 4. The expression profiles of PLS1+ genes were enriched in “axonogenesis,” “Golgi membrane,” “cell junction organization,” “postsynapse,” and “axon guidance.” The top ten significant clusters with their representative enriched terms and gene enrichment network visualization of PLS1+ genes are shown in Figure 5. The corresponding enriched genes for PLS− and PLS1+ are shown in the Supplementary Table 3.

## Discussion

This study analyzed the GMV on T1WI data from CSVD-related cognitive impairment patients and HC individuals to explore the change pattern in GM structure in CSVD-related cognitive impairment. Additionally, the gene expression profiles associated with GMV alterations were examined in CSVD-related cognitive impairment patients using the AHBA database. CSVD-related cognitive impairment patients showed an extensive decrease in GMV in the bilateral temporal lobe and thalamus. Brain structural alterations were closely related to memory and executive function deficits in these patients. A total of 1,564 genes associated with regional changes in GMV in CSVD-related cognitive impairment patients showed an extensive decrease in GMV in the bilateral temporal lobe and thalamus. Brain structural alterations were closely related to memory and executive function deficits in these patients were identified. The enrichment analysis indicated that these genes were ontologically enriched in several terms related to CSVD-related cognitive impairment, such as RNA polymerase II catalytic activity, nucleic acid-binding, aminoacyltransferase activity (negatively correlated with GMV in CSVD-related cognitive impairment), as well as axonogenesis, Golgi membrane, and cell junction organization (positively correlated with GMV in CSVD-related cognitive impairment).

CSVD-related cognitive impairment is a complex condition characterized by a combination of microcirculation disorders and neurodegenerative processes, with structural abnormalities in multiple brain regions and cognitive function gradually worse as the disease progresses. In this work, GM maps of CSVD-related cognitive impairment patients were compared with those of HCs to explore the presence of variations in susceptibility across different structures of CSVD-related cognitive impairment and enhance the understanding of GMV changes in CSVD-related cognitive impairment. Extensive morphological changes were observed in cortical-subcortical substructures, especially in the bilateral temporal lobe and thalamus, for example, bilateral hippocampus, thalamus, parahippocampus, and fusiform, consistent with previous morphometric studies (Liu et al., 2020; Yang et al., 2022). The structure of the temporal lobe, especially the hippocampus, is closely related to cognitive function. Studies reveal the significance of hippocampus as an early pathologically modified area in CSVD patients and its causal impact on brain GMV changes with disease progression (Mu et al., 2024). However, the human cerebral cortex exhibits remarkable cellular diversity, which is critical for its complex functions and overall cognitive abilities. Despite evidence linking brain morphology to cognitive deficits in CSVD-related cognitive impairment, the underlying molecular and cellular mechanisms that induce structural alterations to specific brain regions remain inadequately understood. Thus, new objective techniques are needed to reveal the underlying neuropathological mechanisms of CSVD-related cognitive impairment.

Genetic variants influence neuronal development, synaptic plasticity, and overall brain architecture. An increasing number of genes have been recently associated with CSVD. Approximately 7% CSVD patients were identified as being induced by monogenic causes (Bersano et al., 2016). Notably, mutations in NOTCH3, HTRA1, ABCC6, COL4A1, and COL4A2 (Mancuso et al., 2020; Coste et al., 2021; Guey et al., 2021; Uemura et al., 2023) account for the vast majority of monogenic adult onset of CSVD. Multi-gene sequencing further enhances the understanding of genetic complexity and its implication in health and disease. Transcriptomics enables the integration of genetic association with functional data to decipher the biological roles of genetic risk loci in CSVD-related cognitive impairment. Transcriptome-wide association studies provided the association between one or several genes and CSVD-related cognitive impairment (Zou et al., 2022), enabling the prioritization of putative causal genes for functional follow-up. Advances in technologies and collaborative work have led to substantial progress in the identification of common genetic variants that are associated with CSVD-related cognitive impairment. Several biological processes are involved in the genetic association of CSVD, including the structure and function of the extracellular matrix, myelination, and membrane transport (Bordes et al., 2022). Moreover, some genes associated with CSVD are related to other brain disorders, such as mild cognitive impairment and Alzheimer’s disease, suggesting either mechanistic overlap or genetic pleiotropy (Zou et al., 2022). Genetic predisposition is involved in the individual’s risk of cognitive impairment and the structural integrity of their brain. A total of 782 positively (PLS1+) correlated genomes and 782 negatively (PLS1−) correlated genomes were extracted, composing the regional change in GMV gene list in CSVD-related cognitive impairment individuals.

Gene enrichment analysis showed that RNA polymerase II, catalytic activity acting on nucleic acids, and aminoacyltransferase activity were negatively correlated with GMV in CSVD-related cognitive impairment, meaning that as regional GMV decreased, these processes increased. RNA polymerase II synthesizes messenger RNAs and various non-coding RNAs (including long non-coding RNAs, microRNAs, and small nuclear RNAs) (Girbig et al., 2022), and dysfunctional Pol II affects neuronal gene expression, contributing to cell death and disease progression. Catalytic activity acting on a nucleic acid is referred to the function of enzymes that facilitate the biochemical processes involving nucleic acids, such as DNA and RNA. Aminoacyl transferases are essential enzymes for protein synthesis (Kwon et al., 2019), and a missense mutation in the editing domain of the alanyl-tRNA synthetase in mice results in an intracellular accumulation of misfolded proteins leading to neurodegeneration (Lee et al., 2006). RNA polymerase II, catalytic activity acting on a nucleic acid and aminoacyltransferase activity were mainly involved in the biological processes of nucleic acid metabolism and protein synthesis. Our study showed that these processes were increased in CSVD-related cognitive impairment, probably related to a compensatory action *in vivo*.

Axonogenesis, Golgi membrane, and cell junction organization were positively correlated with GMV in CSVD-related cognitive impairment, suggesting that as regional GMV decreased, these processes also decreased. Axonogenesis, Golgi membrane, and cell junction organization were integral parts to cellular organization, communication, and structural integrity, particularly in the context of nervous system development and tissue function. The Golgi ribbon and the appearance of a dispersed fragmented Golgi were associated with a broad spectrum of diseases including cancer, neurodegeneration, and cardiovascular diseases (Makhoul et al., 2018; Fasimoye et al., 2023). Axonogenesis was determined as possessing multiple connections with neurodegenerative disorders and diseases (Oswald et al., 2017). Our speculation was that in CSVD-related cognitive impairment, Golgi membrane dysfunction affected the biological process of axonogenesis and cell junction organization, leading to GM volume decrease in the cortical-subcortical area. More than 50 independent genetic loci associated with the risk of CSVD have been identified (Bordes et al., 2022), overlapping the results of this study. The gene changes associated with GMV in CSVD-related cognitive impairment provided new insights into the biological mechanisms involved and shed light on the continuum between morphology and the multifactorial nature of CSVD-related cognitive impairment.

The combination of morphotectonics with transcriptomics is essential to capture the complex interplay of factors beyond DNA sequence that lead to CSVD-related cognitive impairment. However, our study has some limitations. Firstly, the amount of data in this study was insufficient for a proper reliability and stability of the results, which therefore needs further confirmation. In the future, the sample size will be increased to perform a deeper investigation into the genes related to the structural and functional changes of CSVD-related cognitive impairment, as well as into the related mechanism at the cellular and molecular level. Secondly, the result of our study cannot be compared and verified with previous studies due to the lack of genetic studies on CSVD-related cognitive impairment structural changes, although our results provide a new reference point for the study of the mechanism regulating CSVD-related cognitive impairment. Finally, the data of the dataset will be used to further verify the results of this study and explore the molecular basis related to other structural and functional changes in CSVD-related cognitive impairment.

## Conclusion

The intricate relationship among cellular diversity, brain morphology, genetic changes, and cognitive impairment in CSVD-related cognitive impairment is a complex but critical area of research. The transcriptome provides insights into this interaction by clarifying the molecular basis of cognitive deficits. Ongoing studies that integrate transcriptomic data with structural and functional imaging will enhance the understanding of how specific brain regions are predisposed to structural alterations and cognitive decline. More work is required to precisely decipher the molecular pathways that underline the observed genetic associations with CSVD-related cognitive impairment, although the existing data provide some preliminary indications. This knowledge could ultimately provide targeted therapeutic strategies aimed at attenuating cognitive impairment in individuals at risk for CSVD-related cognitive impairment.

## Data Availability

The original contributions presented in this study are included in this article/Supplementary material, further inquiries can be directed to the corresponding authors.

## Footnotes

1
http://human.brain-map.org

2
https://www.github.com/netneurolab/abagen

3
https://metascape.org/gp/index.html#/main/step1
