# Supplementary material for: Transcriptional patterns of brain structural abnormalities in CSVD-related cognitive impairment
Source: Front Aging Neurosci. 2024 Nov 29;16:1503806. doi: 10.3389/fnagi.2024.1503806 (PMC11638219; doi:10.3389/fnagi.2024.1503806)
Supplement: Supplementary file 1 [file Table_1.DOCX]

**Supplementary materials**

**Neuropsychological assessment**

Each patient underwent a global cognition and four cognitive domain tests. The four cognitive domain tests contained executive functions, information processing speed, memory and visuospatial functions. Global cognition was assessed using the Mini-Mental State Examination and Montreal Cognitive Assessment. The four cognitive domain tests comprised (i) executive functions: Trail Making Test B, Stroop Color and Word Test (Stroop-C), and Digital Span Test (DST-backward); (ii) information processing speed: Trail Making Test A and Stroop Color and Word Test (Stroop-A, Stroop-B); (iii) memory: Auditory Verbal Learning Test with immediate recall and Auditory Verbal Learning Test with a 20-min delayed recall; and (iv) visuospatial functions: Clock Drawing Test. All raw data were transformed into standard scores (z-scores), which were averaged to assess general cognitive function and other cognitive domains.

Montreal Cognitive Assessment (MoCA) of participants scoring below 26 were classified as cognitive impairment.

**MRI data acquisition**

MRI data for all subjects were acquired from a 3.0-Tesla MR system (Prisma, Siemens Medical Solutions, Inc., Germany). Foam padding was used to minimize head motion. Participants were instructed to stay still, close their eyes, and stay awake during the scan. Three-dimensional T1-weighted images (3D_T1WI) were obtained, followed by the acquisition of T2-weighted imaging (T2WI), T2WI fluid-attenuated inversion recovery (T2WI-FLAIR), susceptibility-weighted imaging (SWI), three-dimensional time-of-flight (3D_TOF) and diffusion weighted imaging (DWI) to identify and remove any subtle brain lesions that were not clinically apparent.

The following parameters were applied: 3D-T1WI scans were acquired at TR=2000 ms, TE=2.49 ms, flip angle (FA)=9, field of view (FOV)=256×256 mm, matrix size=256×256, slice thickness=1 mm, with no gap, and 192 sagittal slices. T2WI, repetition time (TR)=4960 ms, echo time (TE)=109 ms, and slice thickness=5 mm; T2WI-FLAIR, TR=8000 ms, TE=84 ms, and slice thickness=5 mm; SWI, TR=28 ms, TE=20 ms, and slice thickness=2.5 mm; 3D_TOF, TR=21 ms, TE=3.3 ms, and slice thickness=0.7 mm; and DWI data were acquired using a diffusion-weighted echo-planar imaging sequence with TR=6800 ms and TE=75 ms, three b-values at 0, 1500, and 3000 s/mm2 with gradient direction numbers 10, 30, and 60, respectively.

Three senior radiologists with 5, 8, and 12 years of experience in radiodiagnosis diagnosed the disease and ruled out other brain lesions.

**Brain gene expression data processing**

The AHBA ([http://human.brain-map.org](http://human.brain-map.org/)) provides normalized microarray expression data from six donated human brains, including more than 20,000 genes across 3,702 brain tissue samples(1, 2). Because the AHBA dataset includes two right hemisphere data points alone, only the left hemisphere was considered in our analysis(2). The gene expression data was preprocessed using the abagen toolbox([https://www.github.com/netneurolab/abagen](https://www.github.com/netneurolab/abagen)). MRI data from the left hemisphere (from six donors) and the right hemisphere (from two donors) of the AHBA were aggregated across homologous cortical regions to ensure sufficient coverage. The distance between samples was measured using a 2 mm cortical surface distance threshold, and probes were updated to gene annotations using Re-Notator. Only probes with signals significantly greater than the background noise threshold in >50% of samples were retained. Representative probes for genes were selected based on maximum intensity. Gene expression data was normalized using a scaling, outlier-robust sigmoidal normalization method. Finally, after these preprocessing and quality assurance steps, 15633 genes survived. We utilized the Automated Anatomical Labeling(AAL) brain atlas to parcellate the brain to acquire the gene expression matrix. AAL divided the cerebral hemispheres into 116 brain nodes and was spatially matched with 15,633 gene expression profiles to obtain a 116×15,633 matrix.

**Supplementary Table 1** The detailed information on significantly different regions of gray matter volume comparison between the two groups

| Number of voxels | Peak MNI coordinate | Peak intensity | Peak MNI coordinate region(voxels) |
| --- | --- | --- | --- |
| 4006 | -19.5 -33 0 | 8.4045 | Hippocampus_L(677); Thalamus_L(590); ParaHippocampal_L(367); Fusiform_L(351); Thalamus_R(324) ; Amygdala_L (254) |
| 586 | -1.5 34.5 -13.5 | 5.4809 | Rectus_L(263); Frontal_Med_Orb_L(130); Frontal_Med_Orb_R (190) |
| 1426 | 18 -33 1.5 | 8.2401 | Hippocampus_R(540); ParaHippocampal_R(326); Thalamus_R(213); Fusiform_R(147) |
| 434 | -60 -34.5 1.5 | 6.0675 | Temporal_Mid_L(429) |
| 1547 | -39 -22.5 15 | 6.513 | Rolandic_Oper_L(603); Temporal_Sup_L(533); Heschl_L(268) |
| 478 | 45 -13.5 9 | 6.2643 | Insula_R(242); Rolandic_Oper_R(151) |
| 564 | 9 -61.5 12 | 6.4874 | Calcarine_R(408) |

**Supplementary Table 2 The list of genes was selected for enrichment**

| **The top 5% of positively correlated genomes** | **The bottom 5% ofnegatively correlated genomes** |
| --- | --- |
| CEP112 | PTPN1 |
| GFRA4 | AMH |
| RAB3B | NME5 |
| PCOLCE2 | TDRD1 |
| KLK12 | ZSWIM1 |
| MIR4435-2HG | OIP5-AS1 |
| C7orf25 | ELP1 |
| CRYZ | LOC100506388 |
| FAM110B | PLCB1 |
| BEX4 | NUDT22 |
| EFNB1 | TMEM161B-AS1 |
| ARVCF | E2F6 |
| KIAA0319 | CHAF1A |
| CCDC120 | ANXA6 |
| TCEAL1 | EGR2 |
| RBM3 | UBAC2 |
| EAF2 | PPTC7 |
| PBX4 | SETX |
| ASIC2 | EP300 |
| BPGM | UBE3D |
| FRMD3 | PCGF1 |
| WNT4 | POLR2D |
| HIST1H2AB | PXK |
| HDGFL1 | MAVS |
| CCIN | ZNF37BP |
| RRN3 | RNF216 |
| HMGB3P1 | CCNL1 |
| TMEM86A | PNKD |
| UPRT | EDC3 |
| CPSF4 | RRP12 |
| CHPT1 | FASTKD5 |
| SMARCA1 | ZC3H7B |
| PTPRG | SLC25A27 |
| TLL1 | HIRIP3 |
| TFG | WDYHV1 |
| P4HA2 | PIP4P2 |
| GSPT2 | FGD5 |
| LARP1 | POU6F1 |
| ZG16B | PPP2R2C |
| CARMIL3 | RNF115 |
| PLXNC1 | BIVM |
| KLHDC10 | IGLL3P |
| C1QTNF2 | PTH1R |
| ZNF473 | SRRM1 |
| ATRIP | LOC100240734 |
| NCAM1 | MRPS30-DT |
| TRPC4 | RHBDL3 |
| IRF2BPL | ZP3 |
| DNAH14 | FGF14 |
| GPR83 | PER1 |
| EBP | TCEANC2 |
| PLA2G15 | EEF2K |
| PAQR7 | KCNA1 |
| NR2F2 | LOC105377621 |
| SRR | PIAS4 |
| ANO10 | ZNF232 |
| UTP11 | TEFM |
| PSEN2 | TRIM58 |
| NUMBL | METAP2 |
| SEMA5A | KRIT1 |
| WDR5 | EPHB6 |
| HSP90AB1 | COPA |
| DAB1 | ADAM23 |
| TENM3 | MYBL1 |
| KBTBD6 | PIN4P1 |
| SCML1 | CCAR1 |
| PTH2 | LSG1 |
| ZNF764 | COQ3 |
| CCND3 | CCER2 |
| PRR36 | RBM48 |
| CCDC171 | RCCD1 |
| RTBDN | LIF |
| SNTG2 | ELP6 |
| DHRS2 | SPAG9 |
| TSPAN18 | FADS6 |
| FAM212A | ELF2 |
| ZNF280C | PIAS2 |
| KRTAP6-3 | MTDH |
| GHR | SMIM37 |
| ZCCHC17 | DESI2 |
| USP9X | ZNF384 |
| PDGFA | NIF3L1 |
| RRAGB | UIMC1 |
| PGAP1 | MRPL27 |
| MYL12B | RMND5A |
| SPHKAP | LIN9 |
| CIB2 | SUN1 |
| LOC105379807 | MAPK6 |
| AFF2 | GUSBP4 |
| TTC8 | ZNF853 |
| KRTAP17-1 | RNF214 |
| PA2G4P4 | TRAK2 |
| HERC4 | ULK3 |
| CAPZB | PCDH9 |
| C10orf82 | COX17 |
| THSD1 | RAX2 |
| CNPY2 | PNISR |
| P2RX2 | THAP10 |
| ACSL4 | AP1S2 |
| SARAF | EIF5A2 |
| NRP2 | NDRG1 |
| CDC42 | LRRTM3 |
| MRPL32 | LRRC58 |
| PNKP | ART3 |
| CXADRP2 | MYH7B |
| CMIP | GUSBP3 |
| NSUN6 | EGR1 |
| PKIB | NUDT5 |
| C12orf73 | EML2 |
| TRMT112 | TSEN15 |
| ABLIM3 | EPC1 |
| R3HDM4 | DCPS |
| MLNR | CDR2 |
| SMIM14 | SNW1 |
| PSMB4 | DPY19L2P3 |
| TINCR | ANKRD18DP |
| PPP1R3G | URM1 |
| ATP7B | MTO1 |
| CSMD3 | GNL1 |
| NUCB1 | IGIP |
| MARC1 | PCDHB17P |
| PSMB2 | RHOBTB1 |
| NCAN | ZNF664 |
| PANX2 | LRRC38 |
| CABP7 | MED30 |
| ANKRD50 | LOC729218 |
| MCFD2 | ATP5MPL |
| DDX49 | SNRNP35 |
| LUZP2 | RGS5 |
| GPR173 | POLR3D |
| PPP2CB | BBS5 |
| SCUBE2 | SRSF11 |
| PALMD | OR2L3 |
| CALY | TRMT61B |
| LRRC7 | KIF9 |
| ARHGAP6 | DAZAP1 |
| PRMT6 | IDS |
| GMIP | ASAP1-IT1 |
| NPNT | PPM1D |
| EPHA8 | NAP1L1 |
| RFC3 | SEC31B |
| LINC02470 | FBXO33 |
| KIAA0825 | CCL27 |
| ANAPC13 | DEPP1 |
| LOC729732 | TMLHE |
| DNAJB5 | MYL9 |
| IBA57 | NDUFB9 |
| TACR2 | CCNI |
| DYRK3 | LOC100130950 |
| C8orf58 | ILF3 |
| ZNF580 | LONP1 |
| SEMA3D | ARRDC2 |
| ZCCHC12 | ETS1 |
| FAM181A | AIFM3 |
| FAM24B | OXNAD1 |
| TENM1 | BLMH |
| CNGB1 | IFT172 |
| MRPL49 | GTF2IP20 |
| AGPAT1 | KLHL28 |
| MAPK8IP1 | DUSP1 |
| CFAP97D1 | CEMIP |
| PTPRA | LOC101928087 |
| GPR39 | ZNF696 |
| MTMR3 | MCM3AP |
| SLC7A2 | PPP1R9A |
| LRP1B | DDX19B |
| PFDN6 | ABTB1 |
| NDN | CPSF1 |
| JARID2-AS1 | NEIL1 |
| SCN3A | ZSCAN32 |
| ANGPT1 | PRPSAP1 |
| IFI27L1 | PDCL3P4 |
| SCAPER | OXSR1 |
| EPHA5-AS1 | RBBP4 |
| GUF1 | DAPK1-IT1 |
| LMF2 | EPN3 |
| FIBCD1 | SIRT7 |
| VTI1A | ANKS1A |
| RASGEF1A | LZTS3 |
| HELZ2 | OXA1L |
| LIPT2 | AMMECR1L |
| LOC102724156 | UBXN7 |
| DR1 | CNST |
| KCNH2 | TP53TG3D |
| OPHN1 | SLC6A12 |
| SEMA4B | INO80 |
| PIM2 | GSTT1 |
| OCRL | ZNF3 |
| ZDHHC15 | FRMD5 |
| TNNI3 | AKT2 |
| LINC01547 | EDNRA |
| APBA1 | BTAF1 |
| ZWINT | ANKZF1 |
| SQLE | EIF5 |
| ZNF846 | INIP |
| RPP30 | PASK |
| GOLGA3 | SLC35A4 |
| LRRC27 | VN1R1 |
| PSMD13 | MRPS36 |
| NYAP2 | HELB |
| ADRA1A | DDX3Y |
| EBAG9 | COL6A1 |
| FAAP24 | FECH |
| CDC42P3 | MIR9-3HG |
| KIF26B | RAMP3 |
| LRFN1 | ANK1 |
| PTDSS1 | ZSCAN21 |
| AK8 | TRMT2B |
| NKX2-6 | SYT12 |
| RRAS2 | PTPN12 |
| ATP2C1 | ZNF791 |
| BLVRB | HES6 |
| LOC642852 | TAZ |
| CIDEB | NEDD9 |
| NRROS | SLC5A6 |
| COL4A1 | EMC3-AS1 |
| EIF4EBP1 | MYO5C |
| PDCD5 | RASSF1 |
| MYRFL | NUP54 |
| RCN2 | UCP3 |
| RNF215 | OR2L8 |
| POLB | MARK3 |
| CHAC2 | SUGP2 |
| INPP4B | CLASRP |
| TLDC1 | SHD |
| EIF2B3 | RNFT1 |
| PPP1R42 | UBE2G2 |
| ERICH2 | ATAD2 |
| HIGD2B | ATP2B1 |
| FOXI3 | DHX35 |
| PCDH15 | ZW10 |
| TIMP2 | DTNB |
| RECK | GALNS |
| HSDL1 | PCDHB8 |
| FABP1 | PIF1 |
| PVT1 | PSMD6 |
| C1QTNF12 | BAK1 |
| NYNRIN | ATXN1 |
| GASAL1 | BHLHE40 |
| CPNE6 | ZBTB11 |
| GALM | DIS3 |
| SCOC | CDC20 |
| ISG15 | MRPL39 |
| SNCG | CHD4 |
| UBE2L3 | KIAA1551 |
| HHIPL1 | GPATCH8 |
| APP | LOC100286906 |
| NLGN4Y | KCNA2 |
| ARHGDIA | ITPA |
| PPP1R2 | SLC6A8 |
| CLPSL1 | PPA1 |
| TUBB | ZFP14 |
| ADCYAP1R1 | MRPS5 |
| RSU1 | USP36 |
| SPIN4 | PEX6 |
| TP53BP1 | C2CD3 |
| EEA1 | CMC2 |
| PRKG1 | MLLT10 |
| SAMD9 | FBXO32 |
| CLIP3 | NSRP1 |
| PRSS23 | L2HGDH |
| ZNF667 | PAPD4 |
| JMJD7 | SCRT1 |
| LIN7B | LOC101928673 |
| MESP2 | ATG2B |
| DYRK1B | KATNAL2 |
| CTBP2 | GADD45A |
| ATP6V1D | GRAP |
| ZNF518B | FBRS |
| FUOM | C2CD2 |
| CGB1 | NRF1 |
| FAM172A | TARBP2 |
| GADD45GIP1 | TNFRSF10A |
| CADM1 | ITPK1 |
| SIN3A | LRRC59 |
| CYB5B | TTC17 |
| TAS2R60 | SCAMP3 |
| NECAP1 | ANKS6 |
| CALB2 | NR3C1 |
| WDR1 | PRIM1 |
| SLC13A5 | STARD5 |
| SCN7A | CAMKK2 |
| AP2B1 | SKIDA1 |
| FAM71F2 | ISG20L2 |
| LRRTM1 | DPY19L1P1 |
| PGRMC1 | MRGPRF |
| RAB8B | AIFM1 |
| NBDY | NFIX |
| DCHS1 | MCCC1 |
| FAM174A | MARCH2 |
| COPZ1 | VNN2 |
| ZNF285 | LINC00115 |
| DDA1 | SPDYE7P |
| GRM5 | AKAP17A |
| THBD | ASB7 |
| WDR78 | SIRT2 |
| RAC2 | LINC00266-1 |
| CENPM | ARMT1 |
| TAF9B | COG2 |
| GPR176 | RNF144B |
| FAM126A | CIDEA |
| ZNF589 | TSPAN9 |
| ZCCHC18 | ZNF768 |
| DOLPP1 | HNRNPUL2 |
| POLR2L | SMIM3 |
| TTC1 | ARIH2 |
| WDR77 | LOC100287497 |
| TRIM15 | ZMAT4 |
| PIK3AP1 | ABHD10 |
| SLC1A4 | E2F1 |
| PRAF2 | RNF34 |
| SLC35F2 | CCNB1IP1 |
| SHISA7 | ZCCHC4 |
| GPALPP1 | MLXIP |
| ENY2 | AGO1 |
| PCSK5 | LINC01000 |
| CHST9 | MAN2A1 |
| PAFAH1B3 | TAPBP |
| NTN3 | LRRK2 |
| KCTD10 | NOL10 |
| CHD3 | PPARGC1A |
| BBS4 | RING1 |
| DERL3 | STARD10 |
| LPCAT1 | PURA |
| TERF1 | FCHO1 |
| SPA17 | MSANTD2 |
| GYG2 | HOXD9 |
| MRPL55 | ZNF281 |
| NDUFV3 | KIF25 |
| CLCN7 | ENKD1 |
| DRAIC | ATF6B |
| FHL1 | KDM5D |
| FUCA1 | CCDC57 |
| YWHAZ | FOSB |
| USP30-AS1 | RPS6KB1 |
| MANEAL | NAT8L |
| GLRA2 | DNM3 |
| CASK | GSDMB |
| SELENOM | KCTD9 |
| C16orf74 | DDX23 |
| RGR | FAM35A |
| ZNF814 | ELMO3 |
| TM2D2 | NFATC3 |
| HENMT1 | KHDC4 |
| PITHD1 | ZNF700 |
| ARF4 | ZSCAN25 |
| DOCK9 | ABL2 |
| RNASEH2A | MTFP1 |
| ZMYM3 | EXPH5 |
| C9orf131 | RPS6KB2 |
| RAB33B | JMJD1C |
| ARHGAP36 | LINC00999 |
| SLA | SGIP1 |
| ADAMTS7 | ZNF330 |
| NIPSNAP3A | CLCN4 |
| DUSP9 | NOXO1 |
| MRPL44 | RAD54B |
| FRMPD3 | WDR4 |
| SAR1A | MIR29B2CHG |
| CEP83 | UBE2CP5 |
| SCO1 | PPM1B |
| LOC728989 | C6orf106 |
| GNG2 | RASD2 |
| C9orf163 | LOC401357 |
| NDUFB7 | HYDIN2 |
| GOSR2 | C9orf3 |
| TMEM208 | EIF2B2 |
| ZNF579 | ADD1 |
| PLPPR4 | TTI2 |
| DBI | AKAP7 |
| MTMR12 | ZNF655 |
| PCDH18 | AOC2 |
| RRP36 | EVI5 |
| SCN9A | IDH3G |
| GPD2 | ADRB1 |
| SSTR4 | NAIF1 |
| GLOD4 | CDC25B |
| VOPP1 | GPCPD1 |
| FAM105A | NFIC |
| KCNA4 | CNOT6L |
| KPNA3 | ATP6AP1L |
| STARD3NL | SEPT7P2 |
| ZNF449 | CNIH1 |
| RIIAD1 | DLST |
| CPT1C | SORCS1 |
| HNMT | FBXO9 |
| UCK1 | WDR33 |
| STX17-AS1 | SCAF8 |
| FYN | GTPBP6 |
| PLD6 | RNF220 |
| PSMD8 | SLC25A5-AS1 |
| SNX2 | IBTK |
| MIR7515HG | GTF2A1 |
| IFI27L2 | SLC35F3 |
| GPN1 | NUBP1 |
| TOP1MT | SFI1 |
| CFAP73 | ETV6 |
| LINC01176 | KHDC1L |
| ASMTL | TFAP2E |
| NLGN3 | MED12 |
| PTPRS | NR4A1 |
| C1orf52 | SRARP |
| FGFR4 | SNX21 |
| EBPL | ZNF629 |
| C12orf45 | UBAP1L |
| CASD1 | AIP |
| CA8 | NKX1-2 |
| DAPL1 | PPT2 |
| SMAD9 | METTL24 |
| KYAT3 | PIGL |
| FAM9B | ZNFX1 |
| NR2F2-AS1 | DFFB |
| KLHDC8A | CCNQ |
| SSSCA1 | ERRFI1 |
| MRGPRE | SDHAF2 |
| DCLK2 | SLTM |
| SMCO3 | C1RL |
| RPAP3 | RHO |
| CXADRP3 | MTG2 |
| ARHGAP18 | LINC00667 |
| ZC3H3 | ZNF587 |
| PSENEN | CCDC25 |
| FAM161B | POLDIP3 |
| NUTF2 | SLC25A37 |
| VSIG10L | OSTF1 |
| TMEM43 | TAF13 |
| DHCR7 | PIK3R4 |
| ZNRF2 | ZNF143 |
| CROCC | CSTF2T |
| STUM | SYNE1 |
| HTR7 | AQP7 |
| MRPL23 | MED1 |
| ONECUT1 | GABPB1-IT1 |
| AGXT | ITGB3BP |
| OAS1 | STH |
| MRPS25 | RILP |
| DERL1 | DNAJC4 |
| SHC1 | PPIEL |
| SEC22B | CTSZ |
| RAB18 | ADORA1 |
| TXNRD3 | LOC729737 |
| POTEF | WASHC1 |
| BPHL | ATP13A3 |
| CHPF | GTF2H2B |
| SYNGAP1 | PRKCQ-AS1 |
| GID4 | SDSL |
| NAT16 | COL7A1 |
| SDK2 | HRH2 |
| FABP5 | FSTL4 |
| SULF2 | TMEM192 |
| HPGDS | TRPM2 |
| NXN | PLEKHA6 |
| IL13RA2 | CROCCP2 |
| UHMK1 | FTCD |
| RAVER2 | RSAD1 |
| ING5 | SGK494 |
| CCDC170 | SETDB1 |
| ADGRL3 | C22orf46 |
| CPZ | RNF185 |
| FABP5P3 | PCNT |
| DNAJC12 | BBS1 |
| LRP11 | NHLRC2 |
| PEX19 | ZNF609 |
| MBOAT2 | TMEM14B |
| RNF149 | FAM95C |
| TTC23L | ZNF582 |
| GPHB5 | POLH |
| KLHL7 | OSBP2 |
| SCMH1 | SLC25A5 |
| FAM151B | LOC283788 |
| CXADR | TAS2R20 |
| RAB23 | HYDIN |
| PARD6G-AS1 | LNP1 |
| RABAC1 | DDX46 |
| ATP5IF1 | CROCCP3 |
| GGPS1 | PLEKHM1 |
| ZNF219 | CEP192 |
| PALM2 | NFKB1 |
| MAN1B1-AS1 | SNX11 |
| TIPIN | HERPUD1 |
| VGLL3 | TMEM44 |
| DIEXF | CYP1B1-AS1 |
| MAP6 | ZNF562 |
| MYCNOS | PLCXD1 |
| TNKS1BP1 | SUN2 |
| ACRBP | AATK |
| LRRN2 | ZNF267 |
| C20orf144 | NUDT6 |
| CPAMD8 | SLC25A25 |
| LOC100507516 | TMEM86B |
| KLF16 | EIF2S3B |
| HCN4 | SON |
| FAM86B3P | ZBTB38 |
| RBFA | ZGRF1 |
| PLEKHG4B | SPNS2 |
| IGDCC4 | ARHGEF18 |
| FOXA3 | ZNF563 |
| CTXND1 | IMPG1 |
| KLHL5 | SPOPL |
| OR13H1 | ZNF507 |
| OS9 | PARP3 |
| FLVCR1-AS1 | SLC24A2 |
| SLC38A7 | STK11IP |
| TSHB | LARP6 |
| GPR179 | CLPX |
| ENPP1 | VILL |
| VCP | HDDC3 |
| LRRC61 | EIF3A |
| LRCH2 | UBAC1 |
| PPCS | MTMR2 |
| CYTOR | C17orf75 |
| MRPS6 | WDSUB1 |
| DRAXIN | ALDH1A3 |
| TMEM215 | LINC01061 |
| ZDHHC12 | TARDBP |
| VANGL2 | FOXP2 |
| SNAI2 | LRTM2 |
| TNFRSF13C | SIK2 |
| FGD6 | CPLX1 |
| TSG101 | XKR6 |
| PLPBP | MYADML2 |
| PCK2 | ZNF212 |
| GALNT16 | GZF1 |
| SPI1 | MCM8 |
| E2F5 | MPP1 |
| LRRIQ1 | MGAT5 |
| C1orf226 | FES |
| COX6A2 | DEF6 |
| HGH1 | GCSHP3 |
| ZMYND15 | GPR20 |
| PLSCR4 | DDHD2 |
| APLNR | ATG3 |
| MAST2 | PRKCB |
| CCT8 | AAAS |
| LOC389831 | CIT |
| MBLAC2 | ZNF302 |
| PRLH | RASGRP2 |
| ELOB | CIART |
| SCPEP1 | SLC39A13 |
| TMEM61 | COL9A3 |
| CDH2 | SLC38A11 |
| VBP1 | C3orf18 |
| TNNC1 | RBM14 |
| SMARCD3 | ROGDI |
| IL2RB | CACNB4 |
| APRT | LRPAP1 |
| STK17B | FAM78A |
| GTF2IRD2 | RABL6 |
| DOPEY1 | KYAT1 |
| DOC2B | RAD51-AS1 |
| TTL | GPATCH1 |
| SUMO3 | KNG1 |
| CTPS1 | SERTAD4-AS1 |
| LYSMD3 | SAP30 |
| AK4 | LOC220729 |
| FAM117A | LINC00965 |
| LRRC3B | LDLRAD4 |
| DENND6B | FAM76B |
| MEGF11 | PLK1 |
| C3orf67 | TMEM223 |
| ABHD17C | CHD1L |
| GABRA3 | MALAT1 |
| TXNRD1 | DENND4A |
| MAMLD1 | CCNO |
| CCL21 | KLF9 |
| KIF2A | ACTR5 |
| TRAIP | ERCC4 |
| ZNF14 | ST3GAL4 |
| DNALI1 | CKB |
| SLC52A2 | CEP250 |
| ARHGAP5-AS1 | ARMC8 |
| GOLPH3L | ZFP91 |
| C19orf48 | RPS6KA3 |
| PDGFB | PKD2 |
| MED29 | SEMA4C |
| BCLAF3 | CDH7 |
| FAR2P2 | ATG13 |
| YWHAEP1 | FRMD8 |
| PCED1A | LOC101928433 |
| FAM155B | CYR61 |
| KCTD12 | ARHGEF17 |
| TBXA2R | RPL28 |
| POLM | KDM4A |
| IFT74 | IER2 |
| PPM1N | BMS1 |
| CELA2B | TAF1C |
| PPP1R18 | ANKHD1 |
| TMEM181 | MFGE8 |
| ZNF18 | MORC2-AS1 |
| TRIP12 | C12orf49 |
| RXRA | CETN4P |
| SLIT3 | HEXIM1 |
| FSCN2 | PROSER1 |
| RASGRP4 | CNNM1 |
| SPRED3 | FOXP1 |
| DEFB124 | LINC01102 |
| SLAMF7 | ALKBH1 |
| CFP | AFAP1L1 |
| PTGER4 | SRRM3 |
| TLE6 | PAQR3 |
| RPN2 | LINC01158 |
| TMEM234 | KLHL21 |
| C8orf34 | SRP14-AS1 |
| DCAF7 | PAXIP1 |
| FZD7 | QRSL1 |
| LINC01315 | LMLN |
| TLE1 | SERTAD4 |
| CPSF6 | TRERF1 |
| C2orf73 | TM4SF1 |
| SPATA33 | MCTS1 |
| SSC4D | DBP |
| ARL8A | RAPGEF5 |
| SLC26A8 | PCGF6 |
| UFM1 | PNPLA2 |
| GNG10 | SAMD13 |
| SLC7A11 | CDKN1B |
| GCM1 | FDX1 |
| TRIM24 | STX16 |
| AMZ1 | PRAG1 |
| IQCH | EPOR |
| KCTD21 | TMEM38A |
| P2RY12 | ZNF343 |
| MYL5 | DEXI |
| ANK3 | RABEPK |
| CLCN6 | DLG1 |
| ESD | TSEN2 |
| GAN | STARD13 |
| IDH1 | PRMT7 |
| PDZD11 | ASAP2 |
| SPNS3 | POU3F1 |
| CELF6 | LOC100506990 |
| LGR4 | FLCN |
| ME1 | BRWD3 |
| POMP | RRN3P3 |
| ENO1-AS1 | MRPL22 |
| SLC22A4 | REPIN1 |
| TMEM255A | PJVK |
| KLK15 | WDFY3 |
| SSBP1 | ZNF785 |
| ABHD2 | C8orf37 |
| KCTD4 | STK39 |
| FZD9 | ABLIM2 |
| TMEM17 | FIZ1 |
| CCDC90B | ZNF284 |
| LINC00167 | LOC100505938 |
| RGS22 | ZC3H18 |
| ZPR1 | RRS1 |
| TRIM36 | RAD54L2 |
| ME2 | BAZ1B |
| EXOSC7 | LTB |
| DYNLRB1 | PPP3CA |
| SDC3 | AMER1 |
| RRP7A | CWC25 |
| NAV1 | FAT3 |
| RDH14 | TCHH |
| GPC4 | SEPT14 |
| DBX2 | YIPF3 |
| FZD1 | CACNA2D3 |
| SFMBT2 | SEMA3G |
| RAPSN | NFKBIB |
| MATN2 | UBE2C |
| LSAMP | ARAP3 |
| IRX5 | DOHH |
| KRTAP22-1 | GPR158 |
| ADIRF | PPP1R16B |
| PSIP1 | DMKN |
| ACSM1 | DPP3 |
| C2orf70 | HSPA12B |
| SLC29A4 | ADO |
| RNASEH1 | RACGAP1 |
| CCDC112 | MEX3C |
| LTC4S | SCN4B |
| KIAA1211 | IDI2-AS1 |
| FTMT | CCDC47 |
| KARS | HMCES |
| RAB10 | ZNF385B |
| ZCWPW2 | ZNF597 |
| WBP11 | PDE1B |
| MAPK3 | ATL2 |
| FUT7 | DACH1 |
| DAP3 | LINC00963 |
| BIN3-IT1 | JCAD |
| ITPRIPL2 | CDK11A |
| FARSB | TKT |
| MAPK1IP1L | NCAPD3 |
| KLK10 | CDKN2C |
| PRSS21 | ZNF417 |
| HCST | FEZ1 |
| POR | SLC7A5P2 |
| MAPK1 | ANKRD37 |
| CNPY3 | ATP6V1E2 |
| SAMD15 | INSL3 |
| ZNF229 | AURKA |
| DNAAF4 | LGI3 |
| TMA7 | PPP1R3E |
| SEC11C | GPR108 |
| TRAPPC5 | NTHL1 |
| ZNF674 | NEU4 |
| CYP51A1 | DPH3 |
| ANGPT4 | KDM3A |
| ADRB2 | STRN |
| RIOX1 | ZSCAN29 |
| TIMM13 | TBRG1 |
| TFEC | GUCY1A2 |
| CD86 | TNNC2 |
| NUDT17 | ENTPD3 |
| PSMC5 | HSD11B1 |
| TRAP1 | USP2 |
| METTL1 | TRIM26 |
| CTAGE11P | LOC100133331 |
| USP14 | ARHGEF2 |
| SLC2A14 | EIF2B1 |
| NUDT21 | PLN |
| GRIN2B | HAGHL |
| RAB39A | NFS1 |
| CREG1 | SEC14L1 |
| GALNT18 | MLYCD |
| CSF1R | GGT8P |
| CISH | MFN2 |
| LILRA4 | PPP1CB |
| PYGO2 | COL6A2 |
| MRPS10 | AGO2 |
| GABRA2 | LINC01260 |
| MYDGF | RECQL4 |
| INCA1 | TIMM21 |
| WDFY4 | TMEM132C |
| LHX3 | EIF4B |
| PTPRN2 | TGM2 |
| ZBED4 | FAM193A |
| AHDC1 | CALCOCO1 |
| PCBP3 | LOC100190986 |
| DHX58 | MRM1 |
| TGFB2 | LAMA2 |
| KCTD7 | KCNH4 |
| RNGTT | ZNF787 |
| ATP8A2 | HTATIP2 |
| CALU | TTLL11 |
| LPIN3 | MBNL2 |
| CD24 | TRAF2 |
| ZDHHC24 | ACTN4 |
| LOC728715 | TP73-AS1 |
| LINC00842 | FAM49B |
| PPIL4 | GHRLOS |
| CCL17 | MOCS1 |
| AFF3 | TRIM41 |
| FBLN2 | GMPS |
| VMA21 | MTIF2 |
| KRTAP8-1 | ZNF79 |
| MAP3K19 | LINC02145 |
| GNG4 | FUNDC2 |
| MAF | SLC25A41 |
| PRELID3A | SAP30L |
| CSNK1D | SEC14L5 |
| MS4A7 | RARB |
| CHST13 | ONECUT2 |
| PDGFRA | WFDC2 |
| LIPN | FAM133DP |
| WDR25 | TMX2 |
| LOC100130331 | TPBG |
| SIDT2 | MMACHC |
| TTC3P1 | UBQLN4 |
| DHH | BOLA3 |
| ASIP | ZNF576 |
| SP6 | MAD1L1 |
| AKIRIN2 | CDKN2A |
| LINC02381 | FEM1C |
| LINC00158 | POLD4 |
| RAE1 | ZNF106 |
| ZBED6CL | BCORL1 |
| ST8SIA4 | ENPP6 |
| TMEM238 | ZBTB24 |
| IL4I1 | SMURF1 |
| ZNF727 | SLC30A4 |
| TAF10 | PNLDC1 |
| C9orf116 | ZSCAN1 |
| ECHDC3 | SAMD1 |
| DNAL1 | GPX3 |
| ST8SIA2 | POLR3B |
| C6orf118 | INF2 |
| IRF5 | DNAJC27 |
| SNRPD3 | PRUNE2 |
| RHBDD2 | NTPCR |

**Supplementary Table 3 The corresponding enriched genes for PLS1 – and PLS1 +**

| **Description** | **Gene Symbols** | **LogP** | | **Log(q-value)** |
| --- | --- | --- | --- | --- |
| axonogenesis | ANK3,APP,CSF1R,DAB1,EFNB1,EPHA8,FYN,MATN2,NCAM1,NDN,NTN3,OPHN1,PRKG1,SLIT3,LHX3,USP9X,NRP2,SYNGAP1,SEMA5A,NUMBL,PLPPR4,SEMA4B,RAB10,ATP8A2,NLGN3,VANGL2,TTC8,PLEKHG4B,SEMA3D,DRAXIN,POTEF,CDC42,MAP6,SNX2,ST8SIA2,NYAP2,ZPR1,CAPZB,CDH2,SPI1,TGFB2,WDR1,SIDT2,ARF4,BBS4,UHMK1,COL4A1,CSNK1D,AFF2,GRIN2B,PDGFRA,MAPK1,MAPK3,PTPRS,SMARCA1,SSTR4,NR2F2,FZD1,RAB18,SLC7A11,SIN3A,UFM1,WNT4,PCDH18,AKIRIN2,P2RY12,PGAP1,PBX4,PYGO2,NKX2-6,AK8,DCLK2 | -8.174958 | -3.8764772 | |
| Golgi membrane | ARF4,ATP7B,CDC42,FUT7,GOLGA3,NCAM1,PCSK5,PDGFA,PDGFB,CFP,PSEN2,ST8SIA4,ST8SIA2,EBAG9,SEC22B,GOSR2,RAB10,RRAS2,COPZ1,DOP1A,ATP2C1,DHH,ATP6V1D,ATP8A2,RAB39A,GOLPH3L,PSENEN,PANX2,CHPT1,RHBDD2,GALNT16,SCOC,CASD1,CHPF,LPCAT1,RAB33B,CHST9,ZDHHC12,LYSMD3,VTI1A,MANEAL,ZDHHC15,CHST13,GALNT18 | -7.568603 | -3.8764772 | |
| cell junction organization | ASIC2,ANK3,APP,ARHGAP6,CDC42,CDH2,COL4A1,CSF1R,CXADR,GPC4,FYN,GABRA2,GABRA3,GRM5,OPHN1,PTPRS,RAPSN,SNAI2,SNCG,TGFB2,FZD1,FZD9,SYNGAP1,NUMBL,CPNE6,WDR1,NLGN4Y,P2RX2,ADGRL3,RAB8B,NLGN3,SDK2,THSD1,ZDHHC12,LRRTM1,POTEF,SHISA7 | -7.316272 | -3.8764772 | |
| postsynapse | ASIC2,AP2B1,ANK3,APBA1,APP,ARF4,CAPZB,CDC42,CDH2,FABP5,FYN,GABRA2,GABRA3,GLRA2,GRIN2B,GRM5,KCNA4,OPHN1,PTPRS,RAPSN,FZD9,EEA1,NRP2,SYNGAP1,PLPPR4,SEMA4B,NLGN4Y,P2RX2,CADM1,CALY,NLGN3,PALMD,LRRC7,LRFN1,ABHD17C,LIN7B,KCTD12,CPT1C,ZDHHC15,LRRTM1,GPR179,SHISA7,GPC4,VCP,YWHAZ,ST8SIA2,FZD1,PLXNC1,SLC7A11,TUBB,EFNB1,PSEN2,PTPRA,CASK,PSENEN,ZNRF2,MAPK3,ABLIM3,ADGRL3,VTI1A | -7.156947 | -3.8102223 | |
| Axon guidance | CDC42,EFNB1,EPHA8,FYN,MYL5,NTN3,MAPK1,MAPK3,RAC2,SLIT3,TRPC4,SEMA5A,PLXNC1,SEMA4B,ABLIM3,WNT4,MYL12B,SEMA3D,CDH2,SNAI2,NRP2,ARB2A,NCAM1,TGFB2,SP6,APLNR,TAF10,DCHS1,ARHGDIA,FZD1,PDGFA | -5.900074 | -2.7574699 | |
| RNA polymerase II-specific DNA-binding transcription factor binding | ACTN4,ADD1,BBS1,CDKN2A,CHD4,EGR2,EP300,ETS1,GTF2A1,NR4A1,MED1,PRKCB,RARB,STRN,KDM5D,BHLHE40,AIP,PIAS2,MED12,PPARGC1A,SNW1,UBXN7,FOXP1,HES6,TRERF1,KDM3A,NIF3L1,FIZ1,MED30,MTDH,BBS5,SRARP,JMJD1C,E2F1,ERCC4,NR3C1,NFATC3,PER1,PURA,TAF13,BTAF1,CIT,SIRT2,RBM14,CCAR1,CALCOCO1,NFKB1,NFKBIB,SAP30,HTATIP2,PIAS4,BCORL1,SAP30L | -10.21809 | -5.8713676 | |
| catalytic activity, acting on a nucleic acid | CHD4,DFFB,ERCC4,NTHL1,POLH,PRIM1,RBBP4,DDX3Y,ALKBH1,BTAF1,RECQL4,DDX23,CHD1L,DDX46,DDX19B,DIS3,SETX,RAD54L2,RAD54B,AGO2,DCPS,ZCCHC4,INO80,TRMT61B,ANKZF1,QRSL1,ZGRF1,POLR3B,ZNFX1,POLD4,DHX35,NEIL1,MRM1,TRMT2B,PIF1,TSEN2,ISG20L2,MCM8,HELB,PNLDC1,CNOT6L | -7.750287 | -4.035052 | |
| aminoacyltransferase activity | CDKN2A,EGR2,MED1,RING1,TGM2,TRAF2,UBE2G2,TRIM26,PIAS2,KLHL21,MED12,ARIH2,UBE2C,TRIM58,FBXO9,RNF115,RNFT1,MARCHF2,MEX3C,PIAS4,RNF216,RNF220,CCAR1,SMURF1,CCNB1IP1,ATG3,RMND5A,RNF34,ZFP91,UBE3D,MED30,TRIM41,RNF185,WDSUB1,RNF144B,RNF214,BLMH,CDC20,PLK1,UBAC1,KCTD9,FEM1C,URM1,FBXO32,ASB7,FBXO33,SPOPL,DLST,EP300,TAFAZZIN,MCM3AP,SIRT2,PNPLA2,NAT8L,CTSZ,IDS,OXA1L,AURKA,LONP1,HERPUD1,PSMD6,DPP3,CLPX,ARMC8,UBXN7,ANKZF1,ABHD10,UBQLN4,CCDC47,NEU4,UBAP1L | -7.682807 | -4.035052 | |
| chromatin remodeling | CHD4,EGR1,EP300,NAP1L1,PER1,PRKCB,RBBP4,RING1,AURKA,TGM2,KDM5D,PPM1D,ALKBH1,MCM3AP,BAZ1B,BTAF1,DDX23,CHD1L,KDM4A,SETDB1,CHAF1A,RBM14,NUDT5,SIRT2,PAXIP1,RAD54L2,ITGB3BP,RAD54B,ATAD2,SIRT7,UIMC1,PRMT7,INO80,KDM3A,TDRD1,ZNFX1,USP36,ACTR5,EPC1,PCGF6,PCGF1,JMJD1C,MLLT10,NCAPD3,RESF1,SAP30L | -6.906594 | -3.6012619 | |
| DNA damage response | BAK1,CDKN1B,CHD4,GADD45A,E2F1,EP300,ERCC4,GNL1,NTHL1,PLK1,POLH,PPM1D,ALKBH1,BAZ1B,RECQL4,CHD1L,CHAF1A,PARP3,NDRG1,WDR4,SNW1,PAXIP1,SETX,RAD54B,FOXP1,MCTS1,SIRT7,PIAS4,UIMC1,SHLD2,INO80,WDR33,ZGRF1,UBQLN4,HMCES,POLD4,INIP,ARMT1,NEIL1,ACTR5,PIF1,EPC1,MCM8,HELB,ZBTB38,DFFB,NAP1L1,NFIC,NFIX,PRIM1,PURA,RBBP4,LONP1,REPIN1,CCNB1IP1,TBRG1,LIN9,ATF6B,FECH,PTPN1,ARHGEF2,HERPUD1,RNFT1,SEMA4C,SPRING1,TTI2,RNF185,LRRK2,CDKN2A,CIDEA,DACH1,AIFM1 | -6.536607 | -3.3117406 | |


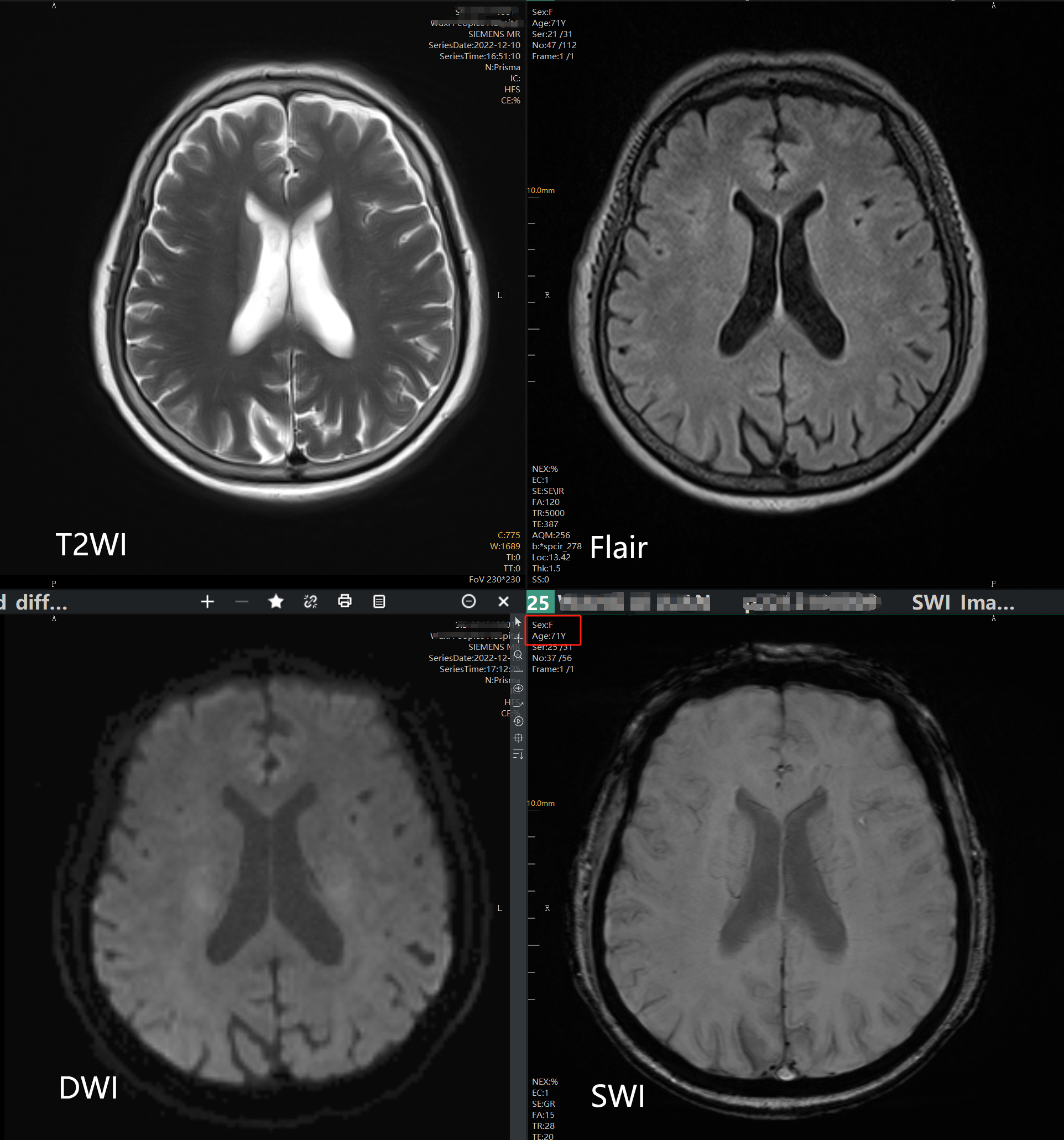


**Supplementary Figure 1 Normal MRI Image of a 71-year-old Female Healthy Control** This image shows the normal MRI findings of a 71-year-old female healthy control. The T2-weighted imaging (T2WI), Fluid-attenuated inversion recovery (FLAIR), Diffusion-weighted imaging (DWI), and Susceptibility-weighted imaging (SWI) sequences all show no abnormalities


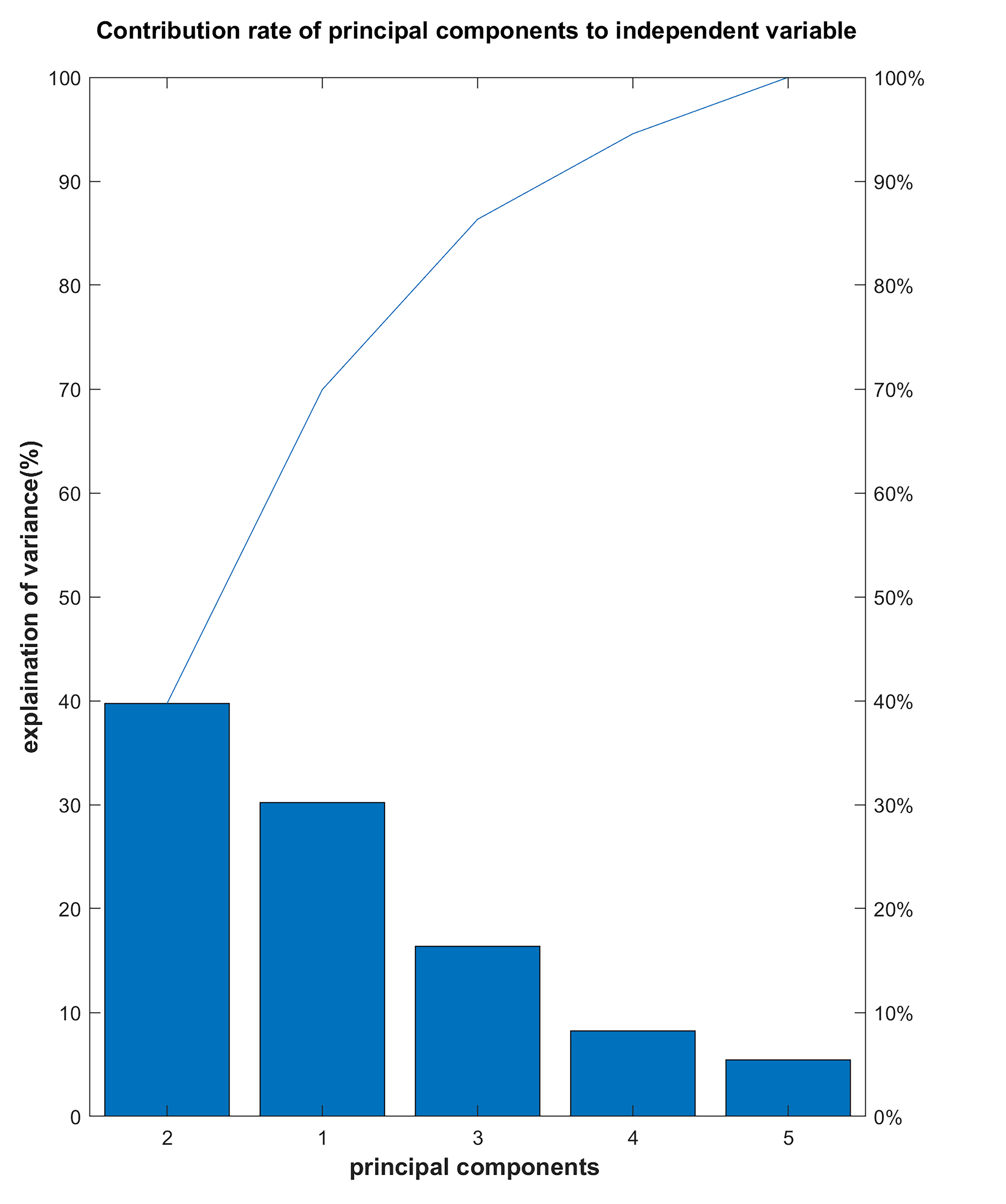


**Supplementary Figure 2 Interpretation of the variance by principal components obtained after PSL dimension reduction**.

The First principal component (PLS1) explained 39.74% of the variance


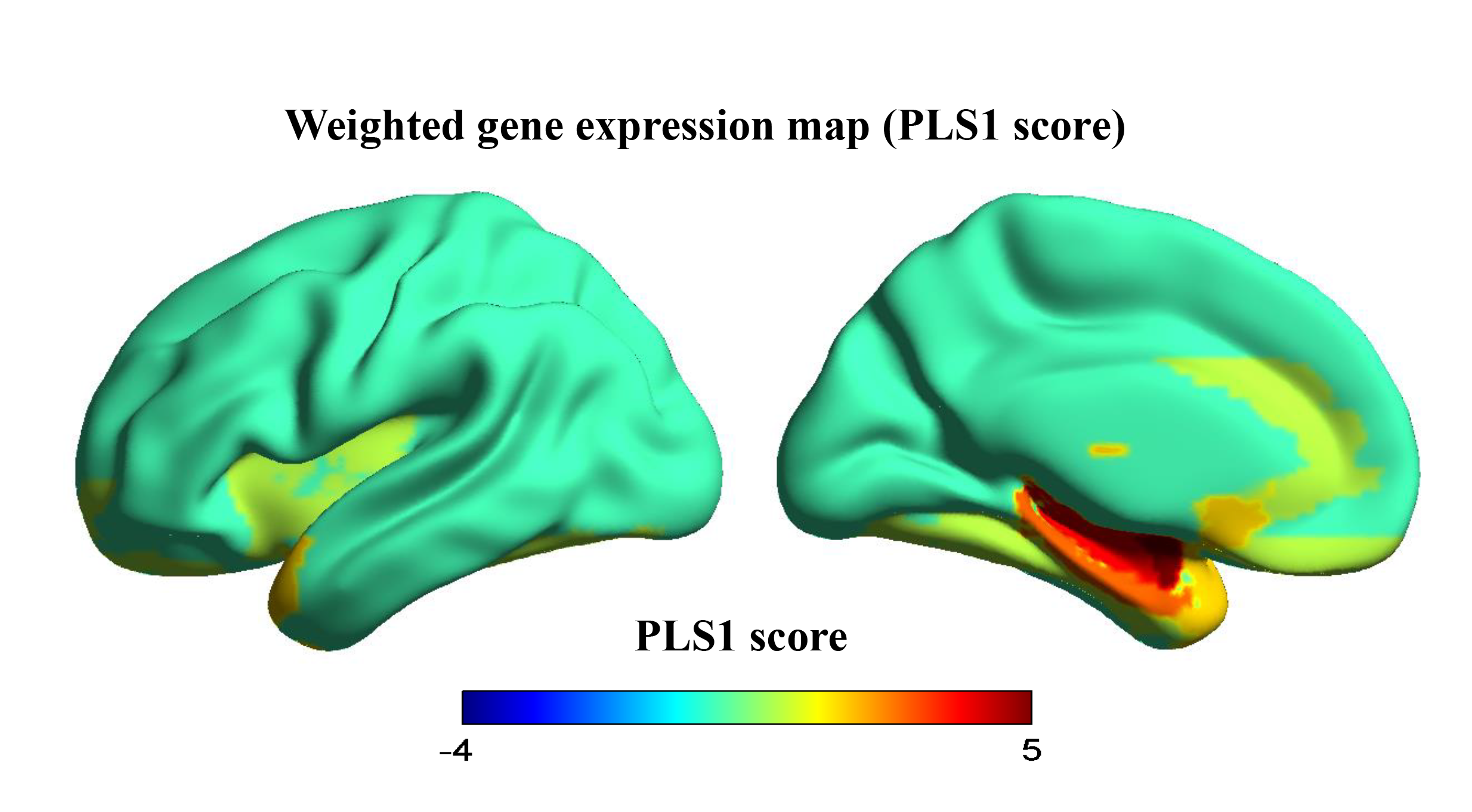


**Supplementary Figure 3 A weighted gene expression map of regional PLS1 scores in the left hemisphere** (Unthresholded)

**
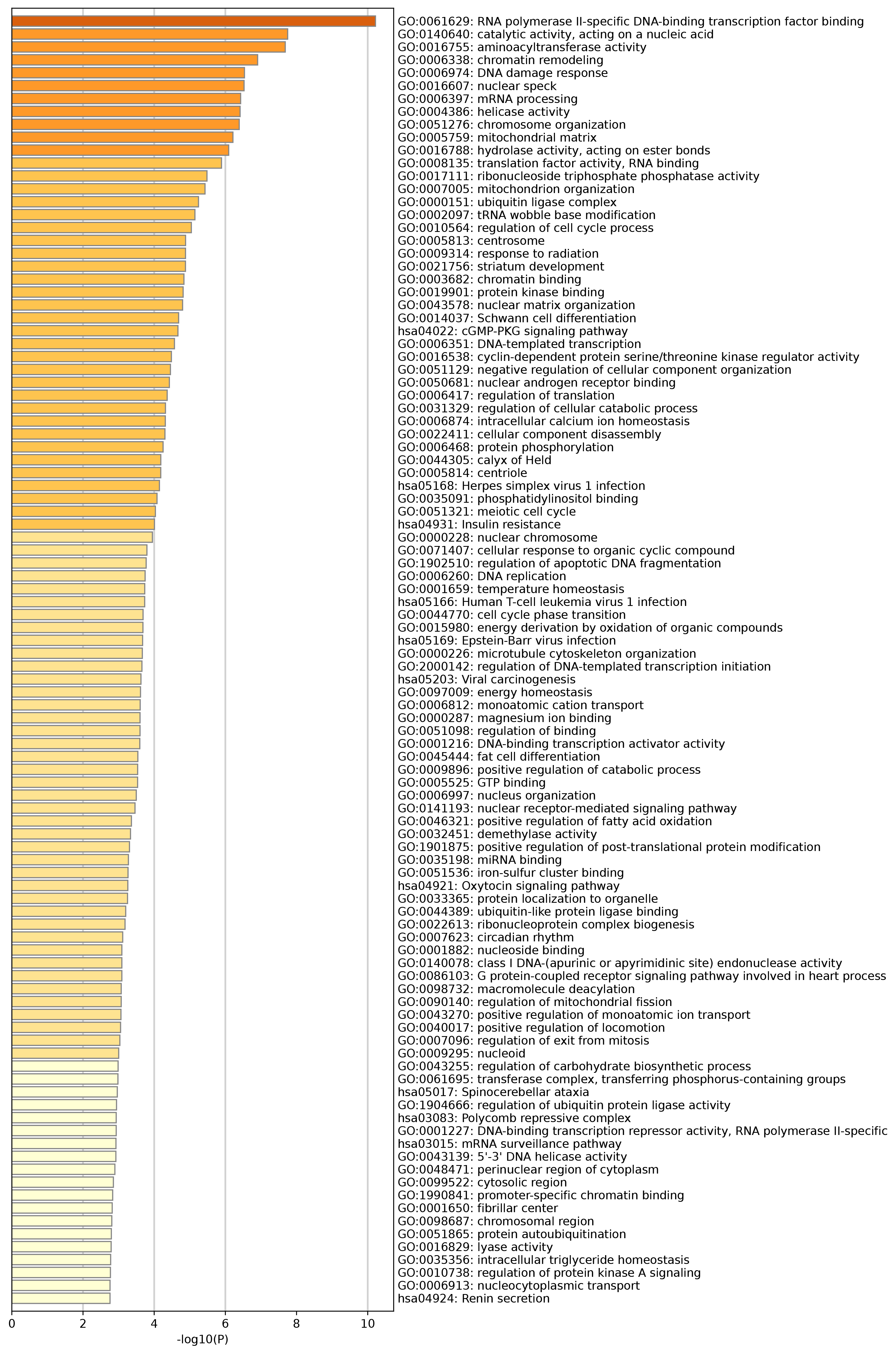
**

**Supplementary Figure 4** The 100 enriched clusters of PLS1-(bottom 5%) genes

**
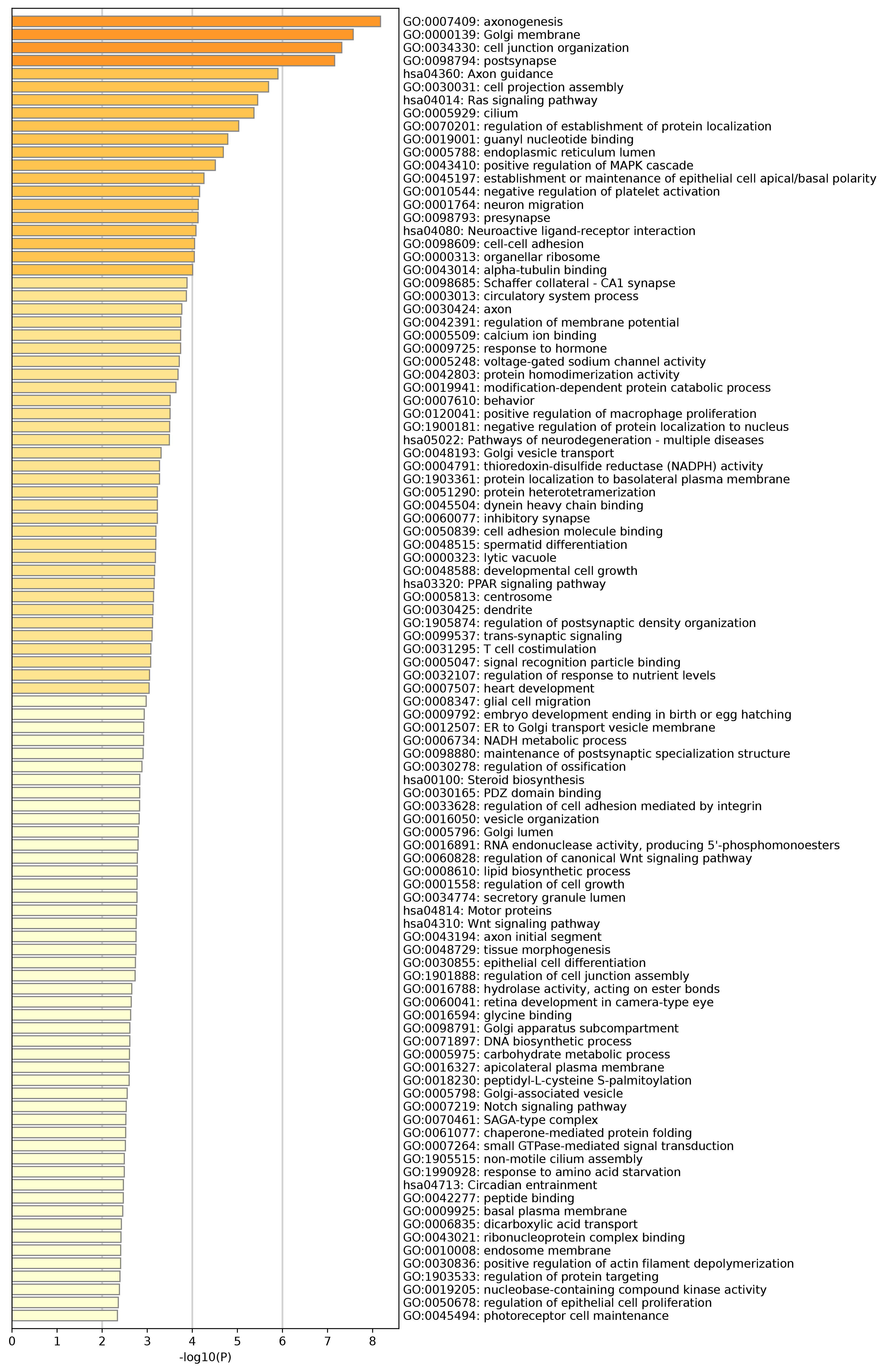
**

**Supplementary Figure 5** The 100 enriched clusters of PLS1+(bottom 5%) genes

**Reference**

1. Qin L, Zhou Q, Sun Y, Pang X, Chen Z, Zheng J. Dynamic functional connectivity and gene expression correlates in temporal lobe epilepsy: insights from hidden markov models. J Transl Med. (2024) 22:763. doi: 10.1186/s12967-024-05580-2

2. Li J, Seidlitz J, Suckling J, Fan F, Ji G-J, Meng Y, et al. Cortical structural differences in major depressive disorder correlate with cell type-specific transcriptional signatures. Nat Commun. (2021) 12:1647. doi: 10.1038/s41467-021-21943-5
